# ﻿Nine new species of *Trigonopterus* Fauvel (Coleoptera, Curculionidae) from Sundaland

**DOI:** 10.3897/zookeys.1124.89318

**Published:** 2022-10-12

**Authors:** Alexander Riedel

**Affiliations:** 1 Museum of Natural History Karlsruhe, Erbprinzenstr 13, D-76133 Karlsruhe, Germany Museum of Natural History Karlsruhe Karlsruhe Germany

**Keywords:** Ancient DNA, Cryptorhynchinae, DNA barcoding, endemism, hyperdiverse, integrative taxonomy, morphology, turbo-taxonomy

## Abstract

The DNA of *Trigonopterus* specimens from the Sundaland region stored between ten and 32 years in museums could be used for next-generation sequencing. The availability of their *cox1* sequence allowed the description of the following nine new species: *Trigonopterusgrimmi***sp. nov.**, *T.johorensis***sp. nov.**, *T.lambirensis***sp. nov.**, *T.linauensis***sp. nov.**, *T.microreticulatus* Riedel, Trnka & Wahab **sp. nov.**, *T.mulensis***sp. nov.**, *T.sarawakensis***sp. nov.**, *T.siamensis***sp. nov.**, and *T.singaporensis***sp. nov.** The alternative original spelling of the name *T.tounensis* Narakusumo & Riedel is chosen to prevail over *T.tounaensis* Narakusumo & Riedel. The new species represent the first country records of *Trigonopterus* for Brunei, Singapore, and Thailand. Thus, the genus´ known area of distribution in the Sundaland region is significantly extended. A key and a catalogue are provided to the *Trigonopterus* species from Borneo, W-Malaysia, Singapore, and Thailand.

## ﻿Introduction

*Trigonopterus* is a hyperdiverse genus of flightless weevils (Curculionidae, Cryptorhynchinae) ranging over the Indo-Australian-Melanesian archipelago. It originated in northern Australia and rapidly diversified in New Guinea (Toussaint et al. 2017). After colonizing Sulawesi, this island acted as a hub for the further dispersal to Borneo, Java, and the Lesser Sunda Islands (Tänzler et al. 2016; Letsch et al. 2020). Currently, there are 489 described species (Riedel et al. 2013b, 2014; Riedel and Tänzler 2016; Narakusumo et al. 2019; Riedel and Narakusumo 2019; Narakusumo and Riedel 2021, and herein), yet a much larger number of undescribed species are at hand. In the following, I report nine new species from the Sundaland region, i.e., from Borneo, Singapore, the Malaysian Peninsula, and Ko Chang Island off the coast of Thailand. These new records significantly expand the genus´ known area of distribution to the west and northwest (Fig. 10). The specimens sequenced for this study were stored ten to 32 years in dry museum collections. Thus, their DNA was somewhat degraded and not suitable for the usual approach of PCR and subsequent Sanger sequencing (Tänzler et al. 2012). However, shotgun sequencing could be used successfully to assemble large portions of the mitochondrial genome (Staats et al. 2013; Yeates et al. 2016). Under the self-imposed premise of describing new species only if some diagnostic DNA sequence data are available (Riedel et al. 2013a) it is now possible to provide names to these species. In doing so, the first records of *Trigonopterus* for the countries Brunei, Singapore, and Thailand are here presented.

## ﻿Materials and methods

This study is based on 46 museum specimens from the Sundaland region. Holotypes were selected from ten specimens for which the *cox1* gene had been sequenced. DNA was extracted nondestructively as described by Riedel et al. (2010) but eluted in only 30 µl of TE buffer. This relatively concentrated template was fully used for library preparation. Genitalia of most specimens did not require extra maceration. They could be directly stained with a 0.01% alcoholic Chlorazol Black solution and stored in glycerol in microvials attached to the pin of the specimens. Illustrations of habitus and genitalia were prepared from holotypes. Type series were supplemented with additional specimens wherever possible. Type depositories are cited using the following codens:

**ANIC**Australian National Insect Collection, Canberra, Australia;

**SMNK** Staatliches Museum für Naturkunde, Karlsruhe, Germany;

**SMNS**Staatliches Museum für Naturkunde, Stuttgart, Germany;

**UBDC** Universiti Brunei Darussalam, Brunei;

**UPOL** Palacky University, Olomouc, Czech Republic.

The methods applied for DNA sequencing differ from our earlier publications as the fragmented DNA of collection vouchers was not suitable for amplifying longer fragments by PCR. Instead, sequencing libraries were prepared with the NEBnext ultra II kit (New England Biolabs, Ipswich, Massachusetts, USA) and tagged with universal dual indexes. Procedures were followed the manufacturer´s protocol except that only half of the recommended volumes were used, i.e., starting with 25 µl of genomic DNA template containing 1.73–18.33 ng DNA. Resulting libraries were quantified using a Qubit 3.0 Fluorometer with the dsDNA HS assay kit (Thermo Fisher Scientific, Waltham, MA, USA). Fragment distribution of libraries was examined using a Fragment Analyzer dsDNA 910 kit (Agilent Technologies, Santa Clara, CA, USA) for a range of 35 to 1,500 bp. Based on the concentration and the average size distribution, the molar concentration was calculated for each sample. The ten samples of this project were pooled in equimolar amounts together with other samples and submitted to Novogene Inc. (Cambridge, U.K.) for sequencing. Libraries of samples ARC1453 (*T.linauensis* sp. nov.), ARC5226 (*T.johorensis* sp. nov.), ARC5227 (*T.singaporensis* sp. nov.) were sequenced using an Illumina Hiseq X 10, while samples ARC7266 (*T.siamensis* sp. nov.), ARC7267 (*T.sarawakensis* sp. nov.), ARC7268 (*T.microreticulatus* Riedel, Trnka & Wahab sp. nov.), ARC7269 (*T.lambirensis* sp. nov.), ARC7270 (*T.grimmi* sp. nov.), ARC7272 (*T.mulensis* sp. nov.) were sequenced on an Illumina Novaseq, in each case for 2 × 150 bp. Reads were processed, assembled and annotated as described earlier (Narakusumo et al. 2020). The *cox1* gene was extracted, used for further analysis, and submitted to GenBank of NCBI (National Center for Biotechnology Information). The accession numbers are provided under each species, e.g., as “(GenBank # OP078703)”. The remaining sequence data generated will be analyzed in future in a wider context. The closest relatives of the species described herein were identified by creating a limited alignment of 23 *cox1* sequences representing 22 species from Sundaland and generating a maximum likelihood reconstruction using the program IQTREE (Nguyen et al. 2015). The uncorrected p-distance was calculated in Geneious Prime 2019.1.3 (Biomatters Ltd, Auckland, New Zealand).

Morphological descriptions are limited to major diagnostic characters as outlined by Riedel et al. (2013a, b). Negative character states (i.e., the absence of a character) are only mentioned explicitly where it appears appropriate. In groups comprising hundreds of species enumerating the absence of rare character states leads to inflated descriptions that distract the reader from the important information, i.e., the diagnostic characters present in a given species.

Morphological terminology follows Beutel and Leschen (2005) and Leschen et al. (2009), i.e., the terms “mesoventrite” / “metaventrite” are used instead of “mesosternite” / “metasternite” and “mesanepisternum” / “metanepisternum” instead of “mesepisternum” / “metepisternum”; “penis” is used instead of “aedeagus” as the tegmen is usually without useful characters in *Trigonopterus* and therefore omitted from species descriptions. Specimens were examined with a Leica M205 C dissecting microscope and a fluorescent desk lamp for illumination. Measurements were taken with the help of an ocular grid. The length of the body was measured in dorsal aspect from the elytral apex to the front of the pronotum. Legs were described in an idealized laterally extended position; there is a dorsal / ventral and an anterior / posterior surface. Habitus illustrations were compiled using a DFC5400 camera adapted to a Z6 APO (all from Leica Microsystems). Photographic illustrations of genitalia were made using a DFC450 camera with L.A.S. 4.8.0 software adapted to an Axio Imager M2 microscope (Carl Zeiss Microscopy), with 5×, respectively 10× A-Plan lenses. Resulting image stacks were compiled using the Helicon Focus 8.1.0 Pro software (Helicon Soft Ltd). For photography genitalia were temporarily embedded in glycerol gelatin as described by Riedel (2005), with their longitudinal axis somewhat lifted caudally, to adequately illustrate structures of the curved down apex. All photographs were enhanced using the program Adobe Photoshop CS6. However, care was taken not to obscure or alter any features of the specimens illustrated.

## ﻿Results

### ﻿ Trigonopterus

Taxon classificationAnimaliaColeopteraCurculionidae

Fauvel, 1862

A447E66E-9A6C-5A36-9B88-B529574ED380

#### Type species.

*Trigonopterusinsignis* Fauvel, 1862, by monotypy.

#### Diagnosis.

Fully apterous genus of Cryptorhynchinae. Length 1.5–6.0 mm. Rostrum in repose not reaching center of mesocoxa. Scutellar shield completely absent externally. Mesothoracic receptacle deep, posteriorly closed. Metanepisternum completely absent externally. Elytra with nine striae (sometimes superficially effaced). Tarsal claws minute. Usually body largely unclothed, without dense vestiture. For additional information, see http://species-id.net/wiki/Trigonopterus.

##### ﻿Descriptions of the species

### ﻿1. Trigonopterus grimmi sp. nov.

Taxon classificationAnimaliaColeopteraCurculionidae

3EFCC42F-C9F1-5E06-8681-059EEB44F1E6

https://zoobank.org/21C702AF-9376-45F0-A3AA-9E6D436467A7

Figs 1 , 10

#### Material examined.

***Holotype*** (SMNS): ARC7270 (GenBank # OP078711), E-Malaysia, Sarawak, Gn. Gading NP, 50–300 m, 20-23-II-2012. ***Paratypes*** (SMNK, SMNS): 2 exx, ARC7271, E-Malaysia, Sarawak, Gn. Gading NP, 50–300 m, 08-10-XII-2010.

#### Diagnostic description.

**Figure 1.**
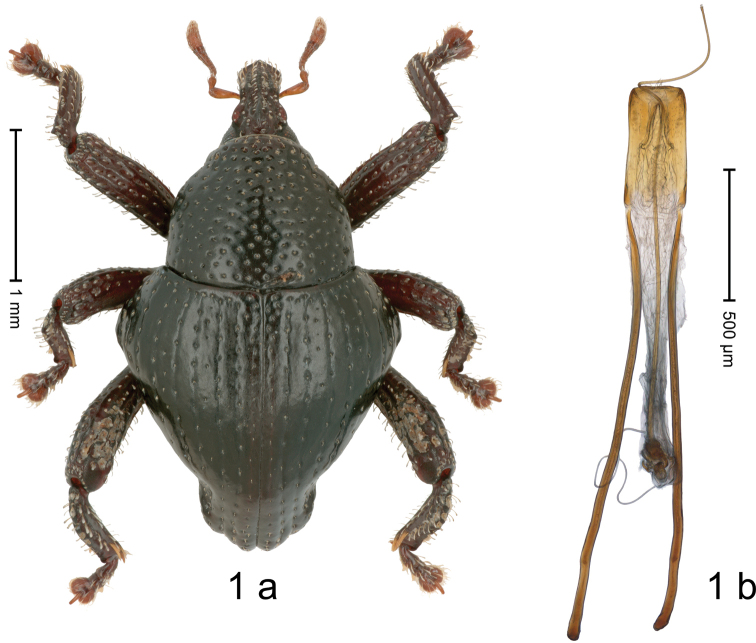
*Trigonopterusgrimmi* sp. nov., holotype **a** habitus **b** penis.

Holotype, male (Fig. 1a). Length 2.90 mm. Color black except antennae light ferruginous, legs dark ferruginous. Body in dorsal aspect subrhomboid, with marked constriction between pronotum and elytron; profile dorsally convex. Rostrum with median and pair of submedian ridges; intervening furrows with sparse rows of erect, clavate scales; epistome with transverse, angulate ridge; forehead in profile with subangulate knob. Pronotum with indistinct subapical constriction; disk densely punctate, interspaces weakly microreticulate; each puncture containing small recumbent seta. Elytra with humeri markedly swollen, laterally subangularly projecting; striae marked by fine lines and rows of small punctures; stria 8 and 9 along humerus each with four to five large punctures; intervals flat, microreticulate; sutural interval with row of minute punctures. Metafemur with dorsoposterior edge denticulate; subapically with stridulatory patch. Dorsal edge of tibiae subbasally dentate. Abdominal ventrites 1–2 forming common cavity, at middle flat, subglabrous, with sparse erect scales; laterally with distinct rim; lateral rim of abdominal ventrite 2 in profile projecting dentiform; abdominal ventrite 5 flat, coarsely punctate-foveate, with sparse erect scales. Penis (Fig. 1b) with sides of body subparallel; apex subangulate; transfer apparatus flagelliform, ca. 4.3 × as long as body; apodemes 3.5 × as long as body; ductus ejaculatorius without bulbus. **Intraspecific variation.** Length 2.70–2.90 mm. Female rostrum dorsally medially subglabrous, sublaterally punctate-rugose; epistome simple. Female elytra with humeri less prominent, convex.

#### Distribution.

Sarawak (Gn. Gading NP). Elevation: ca. 50–300 m.

#### Etymology.

The species is named for the late darkling beetle expert Roland Grimm, who collected the type series of this species. The epithet is a noun in the genitive case.

#### Notes.

*Trigonopterusgrimmi* sp. nov. is coded as “*Trigonopterus* sp. 1247”. This species belongs to the *T.trigonopterus* group. It is closely related to *T.trigonopterus* Riedel, 2014, from which it can be distinguished by its flat elytral intervals and 17.3% p-distance of its *cox1* sequence.

### ﻿2. Trigonopterus johorensis sp. nov.

Taxon classificationAnimaliaColeopteraCurculionidae

BD70C52F-97D1-5039-A155-E2CA1E27437A

https://zoobank.org/494AD9FE-E5EE-439B-BB4C-11E85A24EF23

Figs 2 , 11

#### Material examined.

***Holotype*** (ANIC): ARC5226 (GenBank # OP078705), Malaysia, Johor, Kotta Tingi Falls, 01°50'N, 103°50'E, 100 m, 22-XI-1988, sifted litter.

#### Diagnostic description.

**Figure 2.**
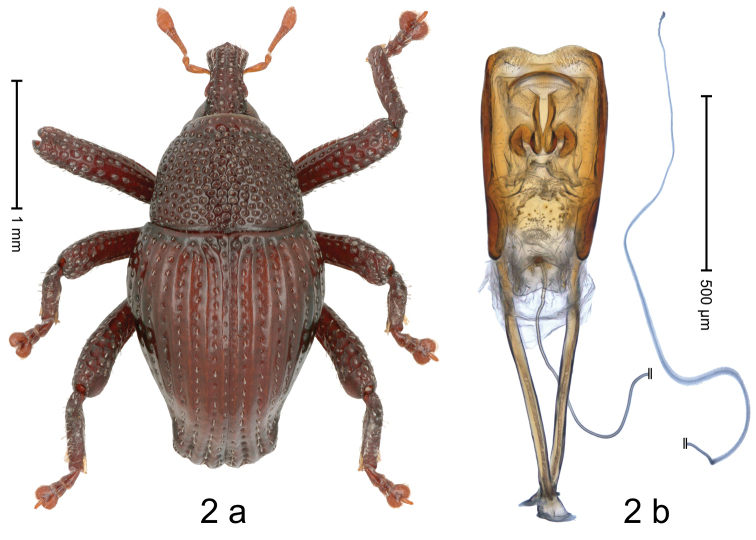
*Trigonopterusjohorensis* sp. nov., holotype **a** habitus **b** penis.

Holotype, male (Fig. 2a). Length 2.88 mm. Color ferruginous. Body in dorsal aspect subovate, with weak constriction between pronotum and elytron; in profile dorsally convex. Rostrum with median and pair of submedian ridges; intervening furrows with punctures and rows of sparse suberect scales; epistome with indistinct subangulate ridge. Pronotum with disk densely coarsely punctate, reticulate; each puncture containing small recumbent scale. Elytra with striae distinct, with small punctures and rows of small suberect scales; intervals costate, subglabrous; elytral apex subtruncate, intervals 1–3 subapically swollen. Femora with crenate anteroventral ridge. Metafemur dorsally scabrous; subapically with stridulatory patch. Dorsal edge of tibiae subbasally dentate. Abdominal ventrites 1–2 forming common cavity, at middle flat, glabrous, laterally and posteriorly with distinct rim; lateral rim of ventrites 1 and 2 in profile projecting dentiform; abdominal ventrite 5 concave, basally with large punctures, apically with smaller punctures. Penis (Fig. 2b) with sides of body slightly diverging; with large anchor-shaped sclerites in apical half; apex bisinuate, with median incision; transfer apparatus small, dentiform; apodemes 1.7 × as long as body; ductus ejaculatorius without distinct bulbus.

#### Distribution.

Malaysia (Johor). Elevation: 100 m.

#### Etymology.

This epithet is a Latinized adjective based on the name of the Malaysian state Johor.

#### Notes.

*Trigonopterusjohorensis* sp. nov. is coded as “*Trigonopterus* sp. 1112”. This species belongs to the *T.attenboroughi* group. It is closely related to *T.attenboroughi* Riedel, 2014, *T.mulensis* sp. nov., and *T.sarawakensis* sp. nov., from which it can be distinguished by the subtruncate elytral apex and 13.7–15.3% p-distance of its *cox1* sequence.

### ﻿3. Trigonopterus lambirensis sp. nov.

Taxon classificationAnimaliaColeopteraCurculionidae

66099929-BB60-5D39-96EE-807AC8310BDD

https://zoobank.org/6D8EAEB1-A045-4309-90FD-0A88D2B1F184

Figs 3 , 11

#### Material examined.

***Holotype*** (SMNS): ARC7269 (GenBank # OP078710), E-Malaysia, Sarawak, 20 km S Miri, Lambir Hill NP, 200 m, 17-18-VIII-2003, sifted. ***Paratype*** (SMNK): 1 ex, same data as holotype.

#### Diagnostic description.

**Figure 3.**
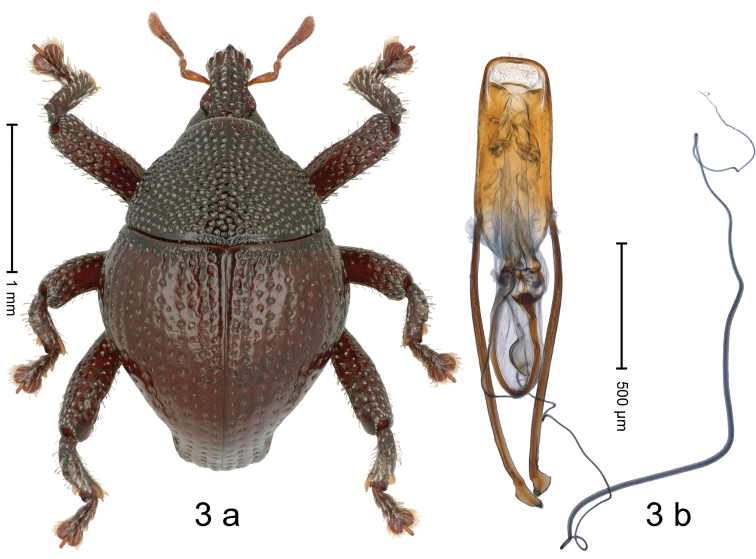
*Trigonopteruslambirensis* sp. nov., holotype **a** habitus **b** penis.

Holotype, male (Fig. 3a). Length 2.55 mm. Color of antennae light ferruginous; legs and elytra dark ferruginous; remainder almost black. Body in dorsal aspect subrotund, with constriction between pronotum and elytron; in profile dorsally convex. Rostrum with median ridge and pair of submedian ridges; median ridge in basal half distinct, terminating at level of antennal insertion; intervening furrows with rows of punctures and erect subclavate scales; epistome with subangulate ridge, at middle with denticle. Pronotum with disk densely coarsely punctate, interspaces reticulate; each puncture containing thin, recumbent seta. Elytra with striae distinct; punctures containing short recumbent seta hardly visible; basal margin bordered by transverse row; intervals flat; sutural interval with row of punctures, coarse near base, minute near apex; other intervals basally with interspersed punctures; stria 8 along humerus with regular punctures. Femora with simple anteroventral ridge. Metafemur with dorsoposterior edge denticulate; subapically with stridulatory patch. Dorsal edge of tibiae subbasally dentate, denticle acute in pro- and mesotibia, blunt in metatibia. Abdominal ventrites 1–2 forming deep common cavity, at middle concave, subglabrous, with sparse erect scales; laterally and posteriorly with distinct rim; abdominal ventrite 5 flat, with coarse punctures, with sparse erect scales. Penis (Fig. 3b) with sides of body subparallel; apex rounded, with sparse setae; transfer apparatus flagelliform, ca. 2.0 × as long as body, coiled, with supporting sclerites; apodemes 1.9 × as long as body; ductus ejaculatorius without distinct bulbus. **Intraspecific variation.** Length 1.95–2.55 mm.

#### Distribution.

Sarawak (Lambir-Hills NP). Elevation: 200 m.

#### Etymology.

This epithet is a Latinized adjective based on Lambir-Hills NP.

#### Notes.

*Trigonopteruslambirensis* sp. nov. is coded as “*Trigonopterus* sp. 1246”. Morphologically it appears related to *T.microreticulatus* Riedel, Trnka & Wahab sp. nov., from which it can be distinguished by the rather polished elytral intervals, the morphology of the penis and 20.9% p-distance of its *cox1* sequence. A comprehensive molecular analysis will need to determine its phylogenetic position.

### ﻿4. Trigonopterus linauensis sp. nov.

Taxon classificationAnimaliaColeopteraCurculionidae

F60707A7-6571-5D4D-AADA-5695B37D0295

https://zoobank.org/4C75E8B6-D47C-4120-8A67-536E38664CE5

Figs 4 , 11

#### Material examined.

***Holotype*** (SMNK): ARC1453 (GenBank # OP078704), E-Malaysia, Sarawak, Belaga, Long Linau, logging camp, ca. 02°45'N, 113°46'E, ca. 400 m, 19-III-1990. ***Paratypes*** (ARC in SMNK): 2 exx, same data as holotype.

#### Diagnostic description.

**Figure 4.**
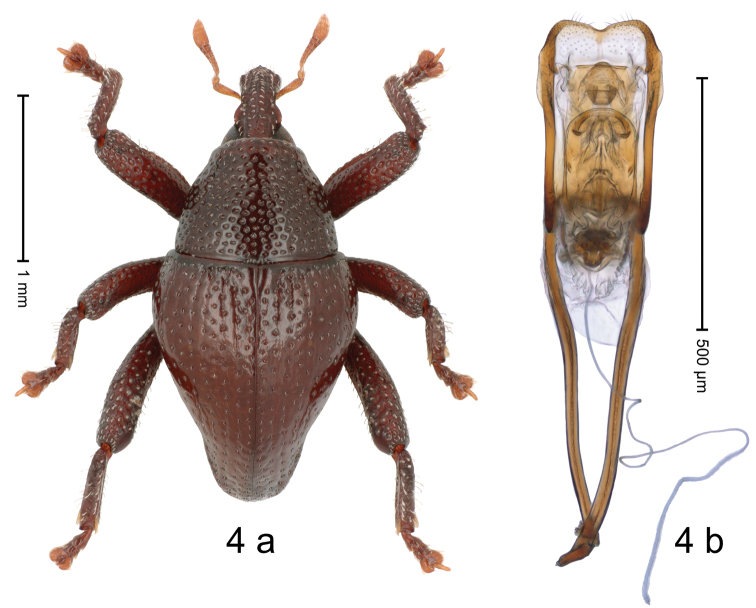
*Trigonopteruslinauensis* sp. nov., holotype **a** habitus **b** penis.

Holotype, male (Fig. 4a). Length 2.28 mm. Color ferruginous, pronotum somewhat darker. Body in dorsal aspect subrhomboid, with weak constriction between pronotum and elytron; in profile dorsally convex, calli at elytral base weakly projecting from outline. Rostrum with median and pair of submedian ridges; intervening furrows with rows of punctures and sparse suberect scales; epistome with indistinct subangulate ridge. Pronotum with disk densely coarsely punctate, reticulate; each puncture containing suberect seta. Elytra with striae marked by hairlines and rows of punctures each containing minute seta; intervals flat, subglabrous, with interspersed punctures, weakly coriaceous; sutural interval at base with glabrous callus. Femora with weakly crenate anteroventral ridge, ending in apical third with small blunt tooth in pro- and metafemur, with minute acute tooth in mesofemur. Profemur in basal third with denticulate posteroventral ridge. Metafemur subapically with stridulatory patch. Dorsal edge of tibiae subbasally dentate, in pro- and mesotibia denticle acute, in metatibia blunt. Abdominal ventrites 1–2 forming deep common cavity, at middle concave, subglabrous, with sparse erect clavate scales; laterally and posteriorly with distinct rim; lateral rim of ventrites 1 and 2 in profile projecting dentiform; abdominal ventrite 5 medially with marked longitudinal impression delimited by distinct ridges, laterally punctate; median depression glabrous, midline with indistinct ridge. Penis (Fig. 4b) with sides of body subparallel, in apical third with narrow lateral flanges; with complex endophallic scle­rites; apex with shallow median incision, with sparse setae; transfer apparatus spiniform, fitted into supporting sclerites; apodemes 1.9 × as long as body; ductus ejaculatorius with indistinct bulbus. **Intraspecific variation.** Length 2.15–2.28 mm. Female unknown.

#### Distribution.

Sarawak (Belaga). Elevation: ca. 400 m.

#### Etymology.

This epithet is a Latinized adjective based on the type locality Long Linau.

#### Notes.

*Trigonopteruslinauensis* sp. nov. is coded as “*Trigonopterus* sp. 1123”. This was the first species of *Trigonopterus* that I personally collected. At the time, I did not fully appreciate the scientific value of the specimens. This species belongs to the *T.attenboroughi* group. It is closely related to *T.sepuluh* Riedel, 2014 and *T.singkawangensis* Riedel, 2014, from which it can be distinguished by the more slender elytral apex, the morphology of the penis and 18.6–19.2% p-distance of its *cox1* sequence.

### ﻿5. Trigonopterus microreticulatus

Taxon classificationAnimaliaColeopteraCurculionidae

Riedel, Trnka & Wahab sp. nov.

82951BC5-B4D7-5048-A763-E00B080CD98E

https://zoobank.org/2D9BCEB3-3E8D-4001-9AD9-0E37F8577777

Figs 5 , 11

#### Material examined.

***Holotype*** (SMNS): ARC7268 (GenBank # OP078709), E-Malaysia, Sarawak, Mulu NP, 100 km SEE Miri, 200 m, 19-24-VIII-2003, sifted. ***Paratypes*** (SMNS, SMNK, UBDC, UPOL): 5 exx, ARC7273, same data as holo­type; 1 ex, ARC4886 (GenBank # OP078703), Brunei, Ulu Temburong N.P., Kuala Belalong FSC, 04°32.793'N, 115°09.450'E, 16-I-2014; 1 ex, Brunei, Ulu Temburong N.P., Kuala Belalong FSC, 04°32.793'N, 115°09.450'E, 06-II-2013; 2 exx, ARC7395, Brunei, Ulu Temburong N.P., Kuala Belalong FSC, 04°32.793'N, 115°09.450'E, 07-II-2013; 4 exx, Brunei, Ulu Temburong N.P., Kuala Belalong FSC, 04°32.793'N, 115°09.450'E, 08-II-2013; 3 exx, Brunei, Ulu Temburong N.P., Kuala Belalong FSC, 04°32.793'N, 115°09.450'E, 10-II-2013; 2 exx, Brunei, Ulu Temburong N.P., Kuala Belalong FSC, 04°32.793'N, 115°09.450'E, 11-II-2013; 2 exx, ARC7396, Brunei, Ulu Temburong N.P., Kuala Belalong FSC, 04°32.793'N, 115°09.450'E, 12-II-2013; 3 exx, ARC7396, Brunei, Ulu Temburong N.P., Kuala Belalong FSC, 04°32.793'N, 115°09.450'E, 13-II-2013; 1 ex, ARC4885, Brunei, Ulu Temburong N.P., Kuala Belalong FSC, 04°32.793'N, 115°09.450'E, 15-II-2013; 2 exx, ARC4887, Brunei, Ulu Temburong N.P., Kuala Belalong FSC, 04°32.793'N, 115°09.450'E, 16-II-2013; 2 exx, Brunei, Ulu Temburong N.P., Kuala Belalong FSC, 04°32.793'N, 115°09.450'E, 16-I-2014.

#### Diagnostic description.

**Figure 5.**
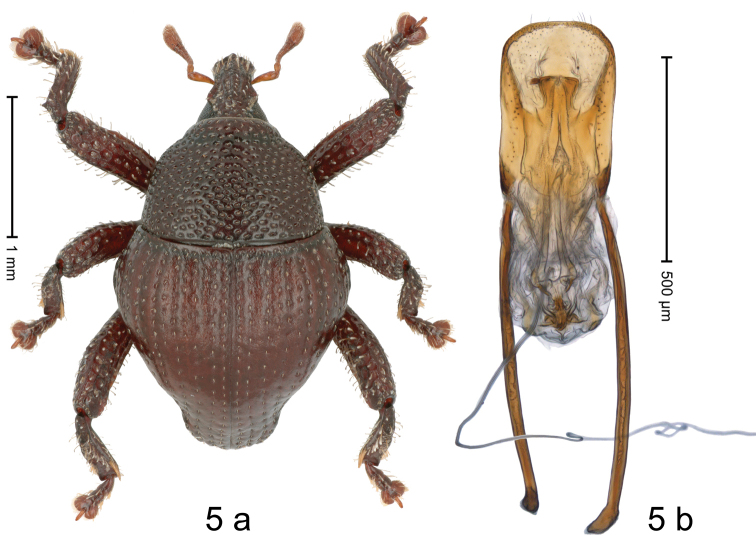
*Trigonopterusmicroreticulatus* Riedel, Trnka & Wahab sp. nov., holotype **a** habitus **b** penis.

Holotype, male (Fig. 5a). Length 2.48 mm. Color of antennae light ferruginous; legs and elytra dark ferruginous; remainder almost black. Body in dorsal aspect subrotund, with constriction between pronotum and elytron; in profile dorsally convex. Rostrum with median and pair of submedian ridges, ending before apex; intervening furrows with rows of punctures and sparse erect scales; epistome with subangulate ridge. Pronotum with disk densely coarsely punctate, interspaces reticulate, microreticulate; each puncture containing small seta. Elytra with striae distinct; punctures each containing short recumbent seta; intervals flat, markedly microreticulate, dull; sutural interval with row of punctures from base to apex; intervals 3–6 with row of punctures in basal half; stria 8 along humerus with three large punctures and four smaller ones. Femora with simple anteroventral ridge. Metafemur with dorsoposterior edge denticulate; subapically with stridulatory patch. Dorsal edge of tibiae subbasally dentate, denticle acute. Abdominal ventrites 1–2 forming deep common cavity, at middle flat, subglabrous, with sparse erect scales; laterally and posteriorly with distinct rim; abdominal ventrite 5 flat, with coarse punctures, basally with sparse erect scales. Penis (Fig. 5b) with sides of body subparallel; apex rounded, with sparse setae; transfer apparatus complex; apodemes 2.3 × as long as body; ductus ejaculatorius without distinct bulbus. **Intraspecific variation.** Length 2.00–2.63 mm. Coloration from light ferruginous to dark ferruginous.

#### Distribution.

Sarawak (Mulu NP); Brunei (Ulu Temburong NP). Elevation: 100–200 m.

#### Etymology.

This epithet is an adjective formed as a compound of the Greek *mikros* (small) plus the Latin *reticulatus* (netted) and refers to the elytral microsculpture.

#### Notes.

*Trigonopterusmicroreticulatus* Riedel, Trnka & Wahab sp. nov. is coded as “*Trigonopterus* sp. 1245”. Morphologically it appears related to *T.lambirensis* sp. nov., from which it can be distinguished by the microreticulate elytra, the morphology of the penis and 20.9% p-distance of its *cox1* sequence. A comprehensive molecular analysis will need to determine its phylogenetic position. It is described under joint authorship with Rodzay Abdul Wahab (Universiti Brunei Darussalam, Tungku, Brunei) and Filip Trnka (Palacky University, Olomouc, Czech Republic).

### ﻿6. Trigonopterus mulensis sp. nov.

Taxon classificationAnimaliaColeopteraCurculionidae

A261DAF0-41BB-54F3-836F-8CD5FCB6BDAC

https://zoobank.org/E34DD151-EC3D-48DE-9330-FD0DEB7BBF5F

Figs 6 , 11

#### Material examined.

***Holotype*** (SMNS): ARC7272 (GenBank # OP078712), E-Malaysia, Sarawak, Mulu NP, 100 km SEE Miri, 200 m, 19-24-VIII-2003, sifted. ***Paratype*** (SMNK): 1 ex, same data as holotype.

#### Diagnostic description.

**Figure 6.**
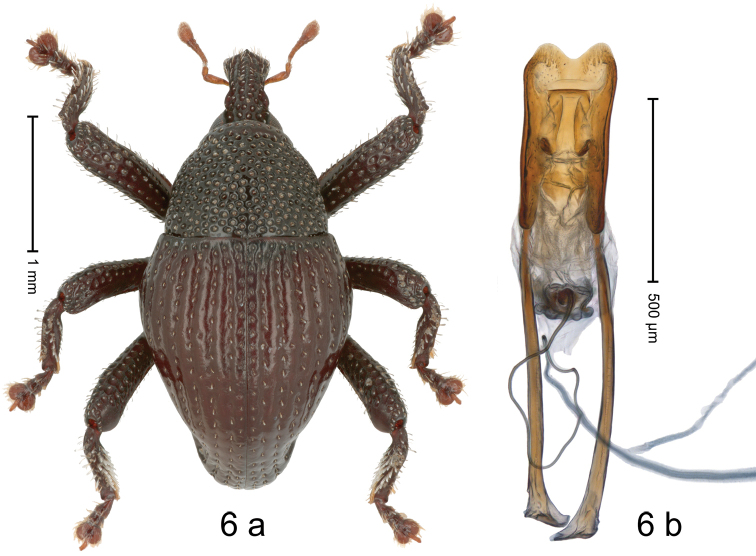
*Trigonopterusmulensis* sp. nov., holotype **a** habitus **b** penis.

Holotype, male (Fig. 6a). Length 2.75 mm. Color of antennae light ferruginous; legs and elytra dark ferruginous; remainder black. Body in dorsal aspect subovate, with weak constriction between pronotum and elytron; in profile dorsally convex. Rostrum with median and pair of submedian ridges; intervening furrows with rows of sparse suberect scales; epistome with median ridge. Pronotum with disk densely coarsely punctate, reticulate; each puncture containing small upcurved scale. Elytra with striae distinct, with small punctures and rows of small suberect scales; intervals costate, subglabrous, coriaceous, sutural interval with row of minute punctures. Femora with simple anteroventral ridge. Metafemur dorsally coarsely punctate; subapically with stridulatory patch. Dorsal edge of tibiae subbasally dentate, in pro- and mesotibia denticle acute, in metatibia blunt; metatibia posteriorly with dense white subclavate scales. Abdominal ventrites 1–2 forming deep common cavity, at middle weakly concave, glabrous, laterally and posteriorly with distinct rim; lateral rim of ventrite 2 in profile projec­ting dentiform; abdominal ventrite 5 at middle with round subglabrous concavity, laterally swollen, punctate. Penis (Fig. 6b) with sides of body subparallel; with large π-shaped sclerite in apical half; apex bisinuate, with median incision; transfer apparatus small, dentiform; apodemes 1.8 X as long as body; ductus ejaculatorius without distinct bulbus.

#### Distribution.

Sarawak (Mulu NP). Elevation: 200 m.

#### Etymology.

This epithet is a Latinized adjective based on Mulu NP.

#### Notes.

*Trigonopterusmulensis* sp. nov. is coded as “*Trigonopterus* sp. 1248”. This species belongs to the *T.attenboroughi* group. It is closely related to *T.attenboroughi* Riedel, 2014 and *T.sarawakensis* sp. nov., from which it can be distinguished by its dense white scales of the metatibia and 9.2–10.3% p-distance of its *cox1* sequence.

### ﻿7. Trigonopterus sarawakensis sp. nov.

Taxon classificationAnimaliaColeopteraCurculionidae

C619FE89-AB80-591F-BB20-DA16858E74D5

https://zoobank.org/F03675AE-22DF-406B-BE14-9C814586378F

Figs 7 , 11

#### Material examined.

***Holotype*** (SMNS): ARC7267 (GenBank # OP078708), E-Malaysia, Sarawak, Mt. Santubong, 17 km N Kuching, 200–400 m, 17-VIII-2003. ***Paratypes***: 1 ex, same data as holotype (SMNK); 2 exx (ARC5228, very low DNA concentration, not sequenced), 11 mi SW Kuching, Semengoh Forest Reserve, leaf-mould berlesate RWT-68.197, 28-31-V-1968 (ANIC, SMNK).

#### Diagnostic description.

**Figure 7.**
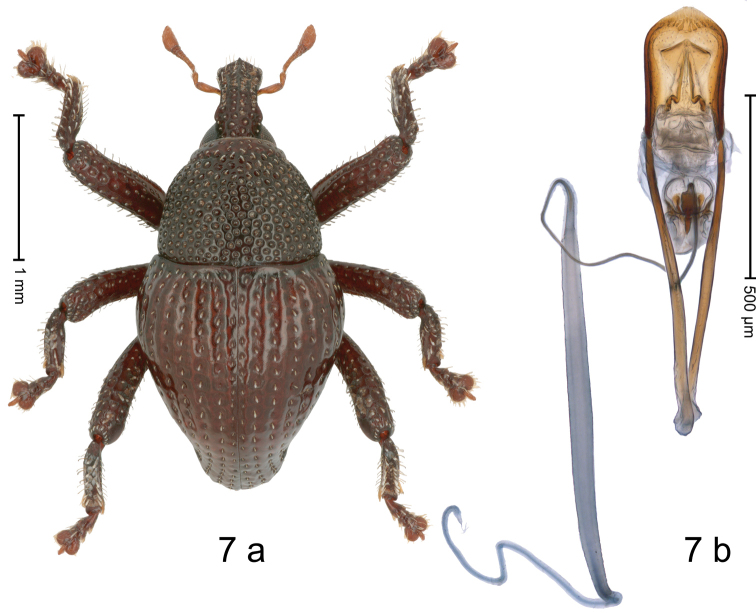
*Trigonopterussarawakensis* sp. nov., holotype **a** habitus **b** penis.

Holotype. Male (Fig. 7a). Length 2.55 mm. Color of antennae light ferruginous; legs and elytra dark ferruginous; remainder black. Body in dorsal aspect subovate, with weak constriction between pronotum and elytron; in profile dorsally convex. Rostrum with median and pair of submedian ridges; interve­ning furrows with rows of punctures and sparse suberect scales; epistome with indistinct subangulate ridge, at middle with minute denticle. Pronotum with disk densely coarsely punctate, reticulate; each puncture containing small suberect scale. Elytra with striae distinct, with coarse punctures and rows of small suberect clavate scales; intervals weakly costate, subglabrous. Femora with weakly crenate anteroventral ridge. Profemur in basal third with weakly denticulate posteroventral ridge. Metafemur subapically with stridulatory patch. Dorsal edge of tibiae with subbasal, blunt angulation. Abdominal ventrites 1–2 forming deep common cavity, at middle weakly concave, glabrous, la­terally and posteriorly with distinct rim; lateral rim of ventrite 2 in profile projecting dentiform; abdominal ventrite 5 medially concave, microreticulate, with sparse punctures, laterally with distinct ridges. Penis (Fig. 7b) with sides of body subparallel; with rhombiform orifical sclerite, medially with pincer-shaped sclerites; apex subangulate; transfer apparatus spiniform; apodemes 2.5 × as long as body; ductus ejaculatorius with distinct bulbus. **Intraspecific variation.** Length 2.38–2.55 mm. Female unknown.

#### Distribution.

Sarawak (Mt. Santubong). Elevation: ca. 200–400 m.

#### Etymology.

This epithet is a Latinized adjective based on Sarawak.

#### Notes.

*Trigonopterussarawakensis* sp. nov. is coded as “*Trigonopterus* sp. 1244”. This species belongs to the *T.attenboroughi* group. It is closely related to *T.attenboroughi* Riedel, 2014 and *T.mulensis* sp. nov., from which it can be distinguished by the subangulate apex of the penis and 8.2–9.2% p-distance of its *cox1* sequence.

### ﻿8. Trigonopterus siamensis sp. nov.

Taxon classificationAnimaliaColeopteraCurculionidae

FC43EE4C-50F8-50EB-ACAB-0D981DDEEFD4

https://zoobank.org/56CE721D-4D2E-446A-B2D9-26034C2082B7

Figs 8 , 10

#### Material examined.

***Holotype*** (SMNS): ARC7266 (GenBank # OP078707), Thailand, Ko Chang, Westseite, 1999.

#### Diagnostic description.

**Figure 8.**
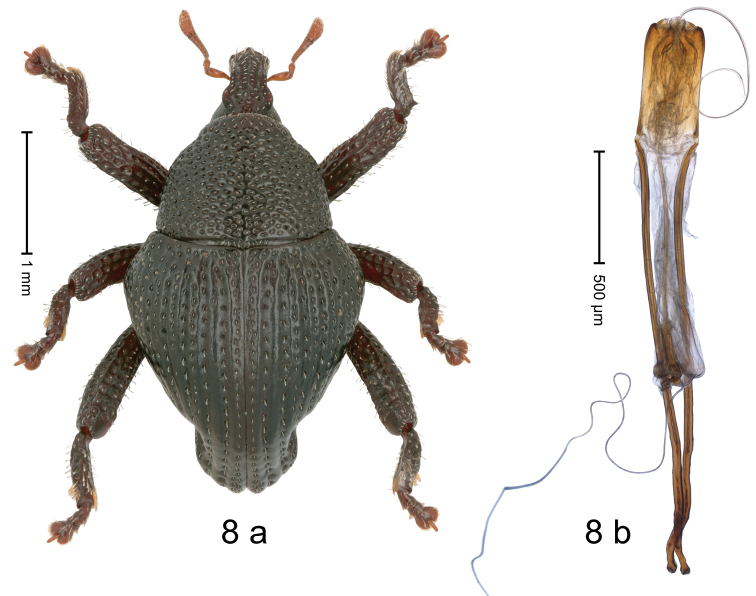
*Trigonopterussiamensis* sp. nov., holotype **a** habitus **b** penis.

Holotype, male (Fig. 8a). Length 3.19 mm. Color black except antennae light ferruginous, legs dark ferruginous. Body in dorsal aspect subrhomboid, with marked constriction between pronotum and elytron; profile dorsally convex. Rostrum with median and pair of submedian ridges; intervening furrows with sparse rows of suberect, clavate scales; epistome with transverse, angulate ridge; forehead in profile with weak subangulate knob. Pronotum with indistinct subapical constriction; disk densely coarsely punctate, interspaces reticulate, weakly microreticulate; each puncture containing small seta. Elytra with humeri swollen, laterally subangularly projecting, coarsely punctate; striae 1–5 distinct, deeply impressed, each with row of coarse punctures and sparse row of suberect scales; intervals costate, microreticulate; sutural interval with row of small punctures; interval 2 almost impunctate; intervals 3–5 with rows of coarse punctures; laterad coarse punctation confused. Metafemur with dorsoposterior edge denticulate; subapically with stridulatory patch. Abdominal ventrites 1–2 forming common cavity, at middle flat, subglabrous, with sparse erect scales; laterally with distinct rim; lateral rim of abdominal ventrite 2 in profile projecting dentiform; abdominal ventrite 5 flat, coarsely punctate-foveate, with sparse erect scales. Penis (Fig. 8b) with sides of body subparallel; apex subangulate; transfer apparatus flagelliform, ca. 3.2 × as long as body; apodemes 3.9 × as long as body; ductus ejaculatorius without bulbus.

#### Distribution.

Thailand (Ko Chang Is.).

#### Etymology.

This epithet is a Latinized adjective based on Siam, the former name of Thailand.

#### Notes.

*Trigonopterussiamensis* sp. nov. is coded as “*Trigonopterus* sp. 1243”. This species belongs to the *T.trigonopterus* group. It is related to *T.singaporensis* sp. nov., from which it can be distinguished by its elytral intervals 3–5 each with a row of coarse punctures and 18.1% p-distance of its *cox1* sequence. It would be important to confirm the locality of this species by additional records.

### ﻿9. Trigonopterus singaporensis sp. nov.

Taxon classificationAnimaliaColeopteraCurculionidae

A66CED17-B44A-564C-9089-712984177B2A

https://zoobank.org/38C610DD-5D33-49BD-A057-CE819E967437

Figs 9 , 10

#### Material examined.

***Holotype*** (ANIC): ARC5227 (GenBank # OP078706), Singapore, Bukit Timah Nat. Res., 01°21'N, 103°47'E, 100 m, 20-XI-1988. ***Paratype*** (SMNK): ARC5172, same data as holotype.

#### Diagnostic description.

**Figure 9.**
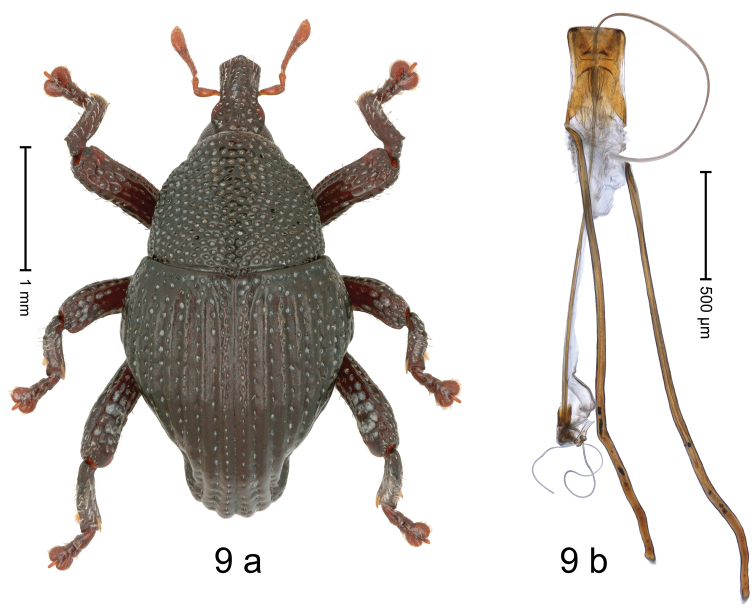
*Trigonopterussingaporensis* sp. nov., holotype **a** habitus **b** penis.

Holotype, male (Fig. 9a). Length 3.20 mm. Color black except antennae light ferruginous, legs dark ferruginous. Body in dorsal aspect subovate, with constriction between pronotum and elytron; profile dorsally convex. Rostrum with median and pair of submedian ridges; intervening furrows with sparse rows of erect, clavate scales; epistome with transverse, angulate ridge; forehead in profile with subangulate knob. Pronotum with indistinct subapical constriction; disk densely coarsely punctate, interspaces reticulate, weakly microreticulate; each puncture containing small recumbent seta. Elytra with humeri swollen, drawn ventrad, in dorsal aspect hardly projecting, rounded; striae distinct, deeply impressed, each with row of small punctures and sparse row of suberect scales; stria 8 along humerus with five large punctures; intervals costate-carinate, microreticulate; sutural interval with denser punctures. Metafemur subapically with stridulatory patch. Dorsal edge of pro- and mesatibia subbasally dentate, metatibia with small denticle. Abdominal ventrites 1–2 forming common cavity, at middle flat, subglabrous, with sparse erect scales; laterally with distinct rim; lateral rim of abdominal ventrite 2 in profile projecting dentiform; abdominal ventrite 5 coarsely punctate, with sparse erect scales, in apical half concave. Penis (Fig. 9b) with body relatively small, sides membranous, appearing weakly concave; apex angulate; transfer apparatus flagelliform, ca. 5.5 × as long as body; apodemes 4.4 × as long as body; ductus ejaculatorius without bulbus. **Intraspecific variation.** Length 3.16–3.20 mm.

#### Distribution.

Singapore (Bukit Timah). Elevation: 100 m.

#### Etymology.

This epithet is a Latinized adjective based on the island of Singapore.

#### Notes.

*Trigonopterussingaporensis* sp. nov. is coded as “*Trigonopterus* sp. 742”. This species belongs to the *T.trigonopterus* group. It is related to *T.siamensis* sp. nov., from which it can be distinguished by its costate-carinate elytral intervals never having rows of coarse punctures and 18.1% p-distance of its *cox1* sequence.

**Figure 10.**
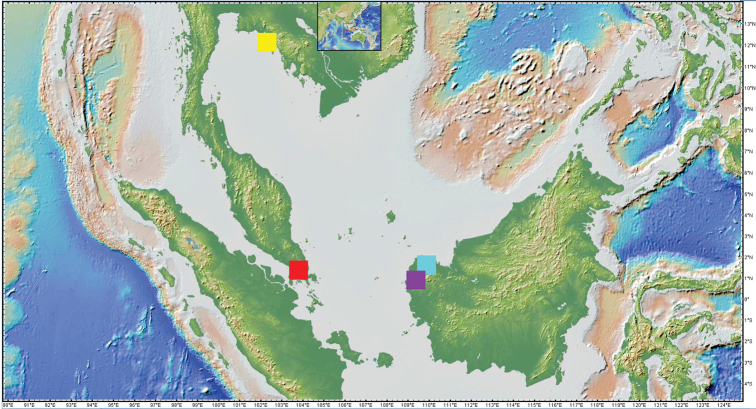
Map of the new *Trigonopterus* species of the *T.trigonopterus* group across the Sundaland area. Prepared using GeoMapApp (www.geomapapp.org; ; Ryan et al. 2009); azure = *T.grimmi* sp. nov., yellow = *T.siamensis* sp. nov., red = " *T.singaporensis* sp. nov., magenta = *T.trigonopterus* Riedel.

**Figure 11.**
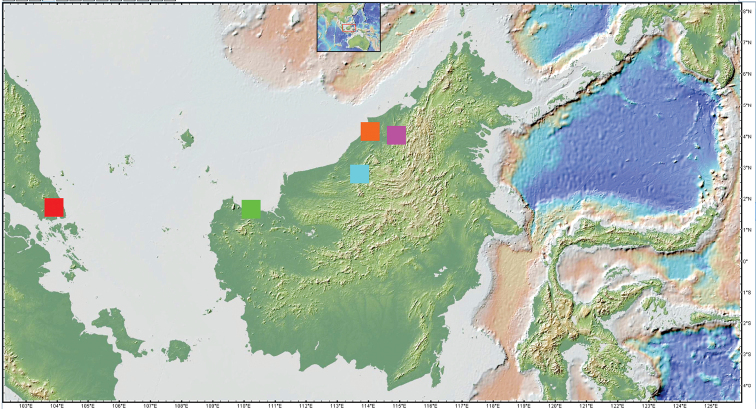
Map of other new *Trigonopterus* species. Prepared using GeoMapApp (www.geomapapp.org; Ryan et al. 2009); red = *T.johorensis* sp. nov., orange = *T.lambirensis* sp. nov., azure = *T.linauensis* sp. nov., magenta = *T.microreticulatus* Riedel, Trnka & Wahab sp. nov. = *T.mulensis* sp. nov., green = *T.sarawakensis* sp. nov.

##### ﻿Remark on *T.tounensis* Narakusumo & Riedel

In the abstract of Narakusumo and Riedel (2021)*T.tounensis* Narakusumo & Riedel was spelled as “*T.tounaensis*”, while in the description and the following figure headings the name *T.tounensis* was used, presenting two alternative spellings. As the first reviser according to article 24.2.1 of the ICZN (International Commission on Zoological Nomenclature, 1999) I decide that the name *T.tounensis* Narakusumo & Riedel should prevail, while “*T.tounaensis*” is to be considered an incorrect alternative spelling.

### ﻿Key to the *Trigonopterus* species from Borneo, west Malaysia, Singapore, and Thailand

**Table:** 

1	Body small, 1.52–1.79 mm. Epistome with dorsoposteriad directed horn and rostrum at middle with dorsal protrusion	***T.wallacei* Riedel, 2014**
–	Body larger, 1.95–3.20 mm. Epistome at most with minute denticle; rostrum with longitudinal ridges but without dorsal protrusions	**2**
2	Pronotum anteriorly with flanges projecting laterally. Anteroventral ridge of meso- and metafemur denticulate	**T.sebelas Riedel, 2014**
–	Pronotum anteriorly simple. Anteroventral ridge of meso- and metafemur simple or crenate, but not denticulate	**3**
3	Elytra with humeri swollen, more or less projecting laterad between mid- and hind leg. Penis with very long apodemes and long flagelliform transfer apparatus	**4**
–	Elytra with humeri simple, not swollen and projecting between mid- and hind leg. Penis with dentiform transfer apparatus (except *T.lambirensis* sp. nov.), usually with additional endophallic sclerites	**7**
4	Elytral striae marked by lines and rows of small punctures, not deeply impressed; elytral intervals flat	***T.grimmi* sp. nov.**
–	Elytral striae deeply impressed; intervals costate or weakly carinate	**5**
5	Elytra ferruginous, humeri markedly projecting	***T.trigonopterus* Riedel, 2014**
–	Elytra black, humeri less projecting	**6**
6	Elytral intervals 3–5 costate, each with row of coarse punctures; punctation of humeri coarse, confused	***T.siamensis* sp. nov.**
–	Elytral intervals costate-carinate, with few small punctures; humeri with re­gular punctation	***T.singaporensis* sp. nov.**
7	Body in dorsal aspect subrotund	**8**
–	Body in dorsal aspect subovate	**9**
8	Elytra dull, microreticulate. Penis with dentiform transfer apparatus	***T.microreticulatus* Riedel, Trnka & Wahab, sp. nov.**
–	Elytra with interspaces between punctures subglabrous. Penis with flagelliform transfer apparatus	***T.lambirensis* sp. nov.**
9	Elytral apex with suture distinctly incised. Abdominal ventrite 2 in profile simple	**10**
–	Elytral apex with suture simple, not incised. Abdominal ventrite 2 in profile projecting dentiform	**11**
10	Penis as in fig. 16b; endophallus containing numerous coarse denticles, apically with pair of relatively small sclerites	**T.bornensis Riedel, 2014**
–	Penis as in fig. 41b; endophallus with complex structures, several sclerites, containing few denticles	**T.kalimantanensis Riedel, 2014**
11	Penis apically with median incision	**12**
–	Penis apically truncate or angulate, without median incision	**15**
12	Body slender subrhomboid. Penis with shallow median incision, with sparse setae	***T.linauensis* sp. nov.**
–	Body subovate. Penis apically bisinuate, with deep incision, without setae	**13**
13	Elytral apex subtruncate. West Malaysia	***T.johorensis* sp. nov.**
–	Elytral apex rounded. Borneo	**14**
14	Metatibia posteriorly with dense white subclavate scales. Penis with large π-shaped sclerite	***T.mulensis* sp. nov.**
–	Metatibia posteriorly with sparse yellowish scales. Penis with endophallic sclerite compressed and folded along middle	***T.attenboroughi* Riedel, 2014**
15	Profemur in basal half posteroventrally with slightly bifid tooth. Male metatibia ventrally with dense fringe of long, stiff setae	***T.santubongensis* Riedel, 2014**
–	Profemur in basal half posteroventrally without tooth. Male metatibia with sparse rows of setae	**16**
16	Base of rostrum in profile simple, evenly confluent with forehead	**17**
–	Median ridge of rostrum swollen in front of forehead, projecting subangularly from profile	**18**
17	Color entirely black. Penis as in Riedel et al. (2014: fig. 77b), apex rounded; ductus ejaculatorius without bulbus	***T.sepuluh* Riedel, 2014**
–	Color of legs and elytra dark ferruginous. Penis (Fig. 7a) with apex subangulate; ductus ejaculatorius with bulbus	***T.sarawakensis* sp. nov.**
18	Penis as in Riedel et al. (2014: fig. 14b), near middle with shallow constriction; transfer apparatus complex, wider than long	***T.bawangensis* Riedel, 2014**
–	Penis as in Riedel et al. (2014: fig. 82b), near middle widened; transfer apparatus complex, small	***T.singkawangensis* Riedel, 2014**

## ﻿Discussion

Specimens of *Trigonopterus* found in two museum collections represented not only new species, but also very notable distribution records. While the hitherto known range of the genus reached its western limit in Lampung province of East Sumatra, the new records from the Malay Peninsula and from a small island off the coast of Thailand extend it considerably to the west and northwest respectively. Since the record from Thailand is represented by a unique specimen and the collector visited Borneo on other trips, there is a small chance of confounded samples. A successful search for additional specimens at the type locality could settle these doubts. However, the biogeographic picture of the *T.trigonopterus* group is one of numerous, genetically divergent species spread over the area of the Sunda shelf. A clade ranging from Borneo to Singapore and the coast of Thailand is not out of the ordinary. Since most of the species from Java and Sumatra (Riedel et al. 2014) were discovered during dedicated field-work focused on the known area of distribution, there is a high likelihood of additional species to be found in north and central Sumatra, the Malay Peninsula, and other parts of Sundaland, areas that have not been searched specifically for these weevils. This emphasizes the value of general museum collections (Fontaine et al. 2012), as they can provide an unbiased overview of larger areas. The past problem precluding their use for molecular analysis was their usually highly fragmented DNA. This could be overcome with next generation sequencing methods (Staats et al. 2013; Yeates et al. 2016). The preparation of the sequencing library and the sequencing itself comes with a cost (currently ~ € 60–70 per sample), but it generates a large amount of sequence data. It is also much cheaper than any dedicated fieldwork to retrieve fresh material. Naturally, not all specimens will work equally well. However, the measurement of the DNA concentration after a non-destructive DNA extraction can quickly clarify the suitability of the material. Library preparation usually works well with as little as 1 ng of input DNA. There is no formal requirement that descriptions of new species need to include sequence information according to the ICZN. However, in a hyperdiverse genus comprising hundreds of similar species, DNA barcodes are of utmost importance to quickly sort and identify species and to avoid producing synonyms. So, under our self-imposed standards that require a fragment of the *cox1* gene for a new description (Riedel et al. 2013a) it was now possible to formally name these interesting new records. The consequent application of this method of generating sequence data from museum specimens will undoubtedly solve many taxonomic issues in future.

### ﻿Provisional catalogue of species groups of *Trigonopterus* in the Sundaland region (except Sumatra and Java)

***T.attenboroughi* group**: *T.attenboroughi* Riedel, *T.bawangensis* Riedel, *T.johorensis* sp. nov., *T.linauensis* sp. nov., *T.mulensis* sp. nov., *T.santubongensis* Riedel, *T.sarawakensis* sp. nov., *T.sebelas* Riedel, *T.sepuluh* Riedel, *T.singkawangensis* Riedel.

***T.bornensis* group**: *T.bornensis* Riedel, *T.kalimantanensis* Riedel.

***T.trigonopterus* group**: *T.grimmi* sp. nov., *T.trigonopterus* Riedel, *T.siamensis* sp. nov., *T.singaporensis* sp. nov.

***T.wallacei* group**: *T.wallacei* Riedel.

**Incertae sedis**: *T.lambirensis* sp. nov., *T.microreticulatus* Riedel, Trnka & Wahab sp. nov.

## Supplementary Material

XML Treatment for Trigonopterus

XML Treatment for Trigonopterus grimmi

XML Treatment for Trigonopterus johorensis

XML Treatment for Trigonopterus lambirensis

XML Treatment for Trigonopterus linauensis

XML Treatment for Trigonopterus microreticulatus

XML Treatment for Trigonopterus mulensis

XML Treatment for Trigonopterus sarawakensis

XML Treatment for Trigonopterus siamensis

XML Treatment for Trigonopterus singaporensis
